# Straintronics and Twistronics in 2D Materials: Structural Control and Predictive Design

**DOI:** 10.1002/smll.75451

**Published:** 2026-09-12

**Authors:** Manoj Tripathi, Sathvik Ajay Iyengar, Hannah J. Wood, Alexander Sredenschek, Md Hasan‐Ur Rahman, Surbhi Slathia, Xinting Shuai, Chandra Sekhar Tiwary, Etienne Z. Gnimpieba, Aditya D. Mohite, Mauricio Terrones, Pulickel M. Ajayan, Venkataramana Gadhamshetty, Vincent Meunier, Alan B. Dalton

**Affiliations:** ^1^ Department of Civil and Environmental Engineering South Dakota School of Mines and Technology Rapid City South Dakota USA; ^2^ Department of Materials Science and NanoEngineering Rice University Houston Texas USA; ^3^ Rice Advanced Materials Institute Rice University Houston Texas USA; ^4^ Department of Physics Pennsylvania State University University Park Pennsylvania USA; ^5^ School of Nano Science and Technology Indian Institute of Technology Kharagpur Kharagpur West Bengal India; ^6^ Department of Chemical and Biomolecular Engineering Rice University Houston Texas USA; ^7^ Metallurgical and Materials Engineering Indian Institute of Technology Kharagpur Kharagpur West Bengal India; ^8^ Biomedical Engineering Department University of South Dakota Sioux Falls South Dakota USA; ^9^ 2D‐materials for Biofilm Engineering Science and Technology (2DBEST) Center South Dakota School Mines Rapid City South Dakota USA; ^10^ Department of Engineering Science and Mechanics Pennsylvania State University University Park Pennsylvania USA; ^11^ Department of Physics and Astronomy University of Sussex Brighton UK

**Keywords:** 2D magnetic materials, machine learning, moiré superlattices, multiscale modeling, strain engineering, straintronics, twist‐angle engineering, twistronics, two‐dimensional materials, van der Waals heterostructures

## Abstract

Strain and twist offer geometric control over lattice reconstruction and electronic symmetry in two‐dimensional (2D) materials. In contrast to chemical doping or compositional alloying, these deformations act on the material response by varying the deformation state and stacking registry. This review explores how the emerging fields of straintronics and twistronics are transforming from isolated demonstrations towards controllable design strategies for electronic, optoelectronic, magnetic, and quantum 2D material systems. Here, we organize a review of recent advances in controlled fabrication, spatially resolved characterization, multiscale modeling, and machine‐learning‐assisted analysis of twist‐ and strain‐induced effects. The examples include carbon‐based nanosheets, transition‐metal dichalcogenides (TMDs), magnetic layers, and layered halide perovskites. Throughout this overview, we emphasize the link from imposed geometric input to local lattice reconstruction and, ultimately, to the measured material or device response. Finally, we identify the main barriers to translating strain‐ and twist‐controlled 2D materials into electronic and optoelectronic devices.

## Graphical abstract

Strain and twist engineering in 2D materials is established as a programmable design parameter responsible for reconstructing lattices, tuning interlayer coupling, and developing new quantum functionalities. Through the integration of advanced fabrication techniques, multiscale characterization, computational modeling, and machine learning, this review underscores a predictive framework that spans discovery‐driven phenomena and strategic engineering approaches for next‐generation devices.

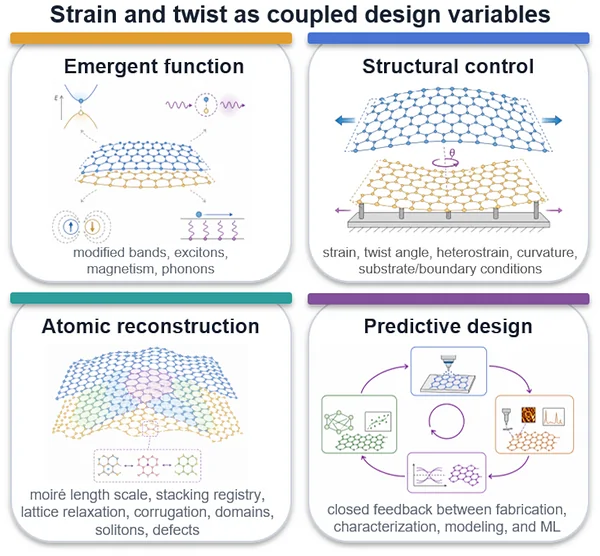

## 1 Introduction

Manipulating and characterizing strain in atomically thin two‐dimensional (2D) materials has emerged as a key avenue for modulation at the nanometer and sub‐nanometer scales. Geometry and orientation can modulate electronic and optical properties, with applications ranging from advanced electronics and optoelectronics to sensing, energy, and quantum technologies. Strain engineering uses controlled deformation (stretching, compression, or bending) to alter band structure, carrier transport, and excitonic response. Atomically thin crystals combine high in‐plane stiffness with anisotropic compressibility and large surface‐to‐volume ratios, making them well suited for probing strain‐driven phenomena at the atomic limit. The accessible strain range is heavily material‐ and geometry‐dependent. Graphene, for instance, shows tensile strains above 10% can be achieved under carefully controlled conditions, a substantially larger value than typical for bulk layered materials before irreversible deformation [1, 2].

Beyond strain, deliberately introducing a twist angle between stacked 2D layers to tune electronic properties, an approach termed twistronics, provides a separate pathway for quantum materials design. Twisting generates artificial moiré superlattices with tunable periodicities that induce modified interlayer hybridization and correlated quantum phases [3]. These moiré superlattices have enabled the observation of moiré excitons, plasmon–phonon polaritons, interlayer magnetism, and 2D ferroelectricity [4, 5]. These phenomena arise from twist‐dependent interlayer coupling and lattice reconstruction, which alter electronic correlation and orbital hybridization.

Strain and twist are often intrinsically coupled in layered 2D systems, where lattice mismatch and rotational misalignment together generate moiré superlattices with complex structural and electronic reconstruction. The moiré period is defined by both quantities; in the nearly matched homobilayer limit, it scales approximately as L_m_ ≈ a/θ, where L_m_ is moiré length, a is lattice constant of the periodic structure, and θ is twist angle. This expression describes the geometric moiré length scale, not a universal scaling of the local strain amplitude [6]. The reconstructed strain field also depends on elastic constants, interfacial adhesion force, stacking‐energy differences, corrugation, defects, and boundary conditions. These effects are particularly prominent in graphene/hBN heterostructures and twisted bilayer graphene, where local stacking, lattice relaxation, and interlayer coupling strongly reshape the electronic structure and correlated behavior.

Together, strain and twist reshape moiré landscapes via lattice relaxation and symmetry breaking. These coupled perturbations can impact electron localization and interaction strength to yield correlated and topological phases in engineered low‐dimensional systems. The diversity of layered materials available for heterostructure assembly, including graphene, TMDs, halide perovskites, and non‐van der Waals crystals, allows access to broad strain‐ and twist‐engineered phenomena [7, 8, 9]. Combining structurally and electronically disparate materials within atomically thin architectures offers a pathway to correlated, excitonic, and hybridized states difficult to access in conventional bulk systems, including chemically bonded and curvature‐driven graphene systems [10, 11].

Understanding these strained and twisted heterostructures increasingly requires multiscale computational approaches, including density functional theory (DFT), molecular dynamics (MD), and continuum modeling, which link lattice relaxation and reconstruction to electronic modulation across moiré systems [12]. Machine‐learning frameworks can speed up prediction and discovery in engineered low‐dimensional materials. These perspectives also connect structure–property control in 2D materials to broader interface‐engineering strategies for energy‐efficient computing [13].

This review considers strain and twist as coupled design variables: we first discuss their physical significance in 2D materials, then examine methods for introducing strain and twist through growth and post‐growth assembly. We then look at experimental characterization, magnetic and optoelectronic case studies, and the role of theory and machine learning to propel the field towards predictive control. The section on nanoscale magnetism highlights stacking‐dependent exchange, while the halide‐perovskite section shows how soft lattices and strongly bound excitons amplify structural perturbations.

## 2 Significance of Strain and Twist in 2D Materials

Strain and twist especially in flexible materials are valuable because they modify electronic structure without requiring chemical tuning. In 2D materials, even small lattice distortions or rotational misalignments can alter band extrema, exciton localization, spin‐valley coupling, interlayer hybridization, and charge transport. These effects allow material properties to be tuned beyond what is typically accessible through chemical doping or surface modification. Local strain engineering in TMDs has been used to control electronic and optical responses, including quantum‐emitter behavior [14]. CMOS‐compatible capping layers have also improved MoS_2_ transistor performance under controlled strain [15].

At the nanoscale, tensile strain, shear strain, curvature, and substrate geometry can substantially modify band structure and interfacial transport, while also generating programmable corrugated structures [11]. Suspended graphene placed over an optimized textured geometry sustains fully recoverable tensile strains of approximately 6% while retaining a Young's modulus near 1 TPa [16]. Under in‐plane shear, graphene undergoes a reversible transition from a flat membrane to periodic wrinkles governed by the device geometry, pretension, and bending stiffness [17]. Suspended transition‐metal dichalcogenides such as MoS_2_ exhibit reversible band‐gap modulation of up to 500 meV under biaxial strains reaching 5.6%, providing a substantial range for tuning optical‐transition energies [18]. Strain in MoS_2_ can modify band structure and carrier injection. In WS_2_, spin–valley relaxation is strongly tied to electronic and phonon structure, which is relevant for strain‐based valleytronic design [19]. Strain‐tunable band gaps can also broaden optical absorption from the visible to near‐infrared range, while photodetectors have shown enhanced responsivity under applied strain [20, 21].

In twisted 2D systems, moiré superlattices strongly reconstruct the electronic band structure and can generate “flat bands,” where reduced carrier velocity enhances electron–electron interactions. In graphene‐based systems, these effects become especially pronounced at small twist angles of approximately 1–3°, where interlayer coupling strongly modifies transport and correlated behavior. The archetypal example is twisted bilayer graphene near the “magic angle” of 1.1°, which exhibits unconventional superconductivity [22], correlated insulating states [23], and Kondo physics [24]. Twisted bilayer graphene also exhibits van Hove singularities, corresponding to sharp peaks in the electronic density of states that can promote magnetic and correlated electronic instabilities [25, 26]. Beyond graphene, twisted van der Waals heterostructures with broken inversion symmetry can host ferroelectric domains with twist‐dependent polarization textures [27], while TMD moiré systems exhibit spatially varying band landscapes that can localize excitons [5, 28].

Strain and twist therefore provide complementary approaches for engineering band structures and correlated‐excitonic behavior in low‐dimensional materials, while also enabling access to emergent condensed matter phenomena and new device architectures for electronic and optoelectronic technologies.

## 3 Applying Strain and Twist to Stacked 2D Materials

Translating straintronics and twistronics into device‐relevant structures requires fabrication routes that control local strain fields, twist angle, interface cleanliness, and lattice relaxation. These structures can be formed during growth, where strain and twist emerge in situ, or through ex situ conversion and transfer‐based stacking. Figure 1A summarizes top‐down and bottom‐up pathways for introducing strain and twist [29, 30, 31]. Deterministic transfer methods bridge these strategies by enabling controlled stacking and alignment across diverse material systems. As fabrication moves toward scalable manufacturing, increasing emphasis is being placed on automation, and data‐assisted characterization [32].

**FIGURE 1:**
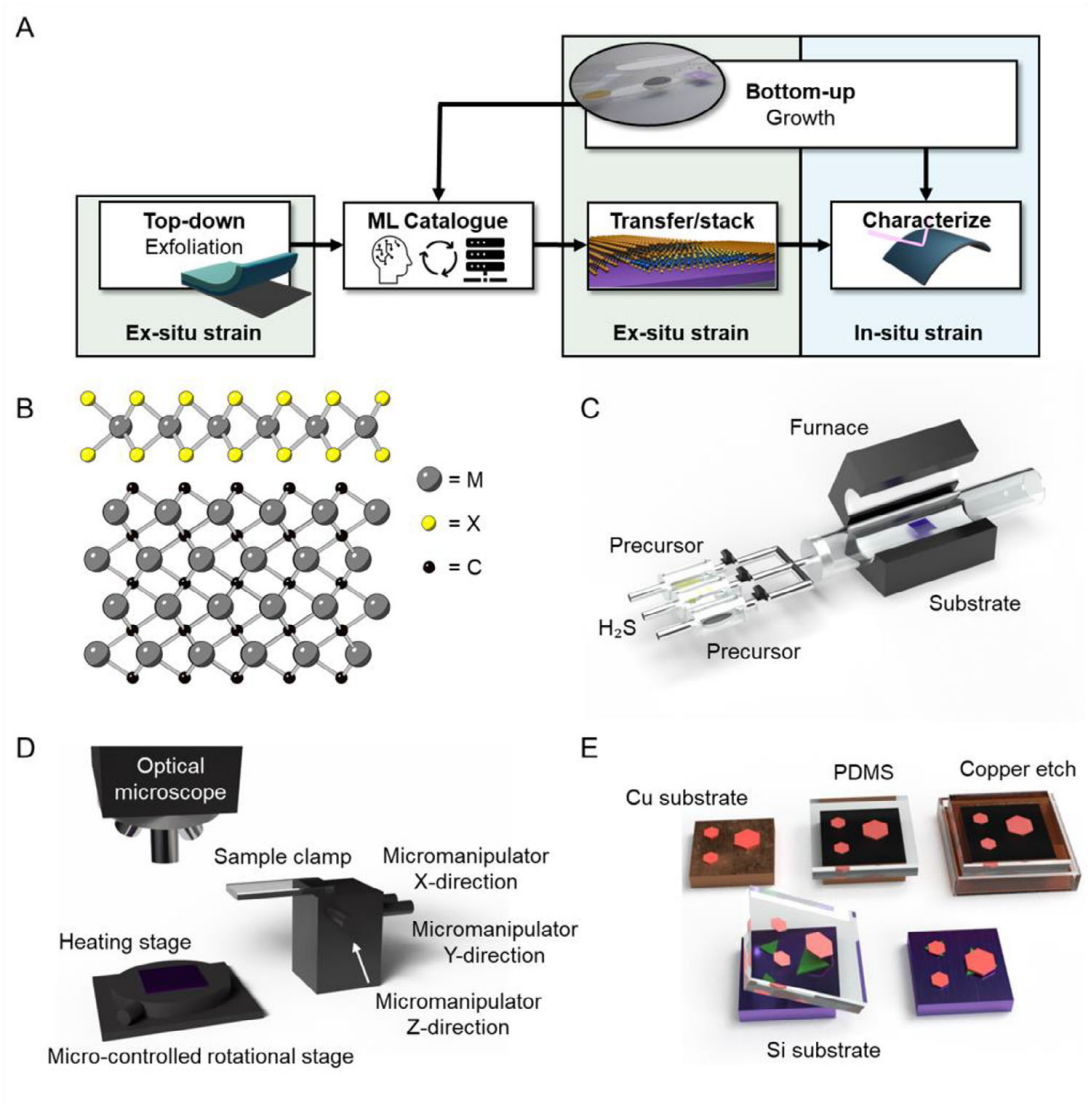
Experimental routes for generating strain‐ and twist‐controlled heterostructures. (A) Workflow connecting top‐down exfoliation or patterning, bottom‐up growth, transfer and stacking, structural characterization, and machine‐learning‐based cataloging of material configurations and properties. (B) Schematic of a representative vertical 2D/3D heterostructure. (C) Two‐step reactive conversion in a tube furnace using an H_2_S‐containing precursor stream. (D) Deterministic‐transfer platform comprising an optical microscope, heating stage, microcontrolled rotational stage, and x–y–z micromanipulators. (E) Semi‐dry transfer sequence using a PDMS support, Cu‐substrate etching, rotational alignment, and placement onto a Si substrate. (A, B) Adapted from [29, 30, 31]. (C) Adapted from [33]. (D, E) Adapted from [34, 35].

The trade‐off between top‐down and bottom‐up methods motivates research into more controlled strain and twist engineering. Ex situ methods can locally pattern surface topography, while in situ growth can tune the chemistry or defect density of a seed monolayer before stacking or overgrowth. Controlled defects, surface chemistry, substrate steps, and spatially defined nucleation sites may influence where a second layer forms and how it relaxes locally. Such methods can improve orientation control, but deterministic twist‐angle control remains strongly system‐dependent. Scalable patterning may eventually extend this control over larger areas.

A major advantage of in situ heterostructure growth is the formation of cleaner interfaces with reduced contamination and interfacial disorder. Chemical vapor deposition (CVD) remains one of the most widely used methods for producing both lateral and vertical heterostructures in situ, including the representative vertical van der Waals heterostructure shown in Figure 1B. Although precise twist‐angle control remains challenging in CVD‐grown systems, growth temperature and precursor engineering, including sequential nucleation control, have improved alignment in selected systems [36, 37, 38].

## 4 In Situ Synthesis and Growth

Effective formation of strain‐ and twist‐engineered heterostructures by CVD relies on precise control over growth temperature, precursor chemistry, gas‐flow dynamics, and nucleation sequence [39, 40]. Substrate engineering and precursor spatial distribution can also influence interlayer alignment, strain distribution, and the formation of lateral or vertical heterostructures [41, 42]. Together, these factors govern interface quality and crystallographic registry, which set the resulting moiré reconstruction in stacked systems.

In situ growth approaches have led to the synthesis of both twisted multilayer and vertically integrated heterostructures across a broad range of material systems. In graphene‐based systems, low‐pressure CVD growth can produce twisted multilayer graphene (tMLG) with characteristic twist‐angle distributions spanning 3–30° [43, 44]. More broadly, precursor confinement and sequential or multistep growth strategies enable the controlled formation of vertical heterostructures such as MoO_3–_MoS_2_ systems, where interfacial stacking and growth order strongly influence the resulting architecture [45, 46]. Sequential growth approaches are particularly useful for minimizing interfacial degradation while promoting controlled overgrowth between dissimilar materials.

Beyond vertical stacking, CVD can also form lateral heterostructures through spatially selective epitaxy and controlled precursor switching [36]. Multistep growth processes, including reverse gas‐flow and temperature‐modulated schemes, produce laterally stitched chalcogenide heterostructures with sharper interfaces and reduced thermal degradation. These methods offer additional ways to engineer local strain fields and interfacial electronic structure across atomically thin architectures.

Machine learning and automated analysis are increasingly used to correlate growth parameters with post‐growth optical and structural measurements. This can improve reproducibility and accelerate exploration of large process spaces [32]. Most studies, however, still use data‐driven analysis after growth rather than a closed‐loop CVD system that adjusts precursor delivery and furnace conditions in real time.

## 5 Ex Situ Conversion

Heterostructure generation through ex situ conversion is typically performed after synthesis of the parent material by methods such as CVD, mechanical exfoliation, liquid exfoliation, or physical vapor deposition. This approach enables access to hybrid structures and compositional architectures that are often difficult to realize through direct growth alone [47]. Conversion is commonly achieved using reactive gas environments such as H_2_S or NH_3_, as shown in Figure 1C [33, 48, 49], although temperature‐controlled and precursor‐limited conversion pathways have also been demonstrated, particularly in oxide‐based systems [50]. Unlike direct growth approaches, conversion‐based methods rely on partial chemical transformation of an existing lattice, making interface evolution strongly dependent on local reactivity and diffusion pathways, often mediated by defects. As a result, conversion frequently initiates at edges, grain boundaries, and defect‐rich regions, where structural reconstruction and chemical substitution are energetically favored [48, 51]. These processes can generate substantial local strain because the parent and converted domains differ in crystal structure and surface termination. Consequently, achieving atomically sharp interfaces remains a central challenge in conversion‐based heterostructuring.

For example, MoS_2–_MoN lateral heterostructures formed through epitaxial atomic substitution demonstrate that controlled exposure of exfoliated MoS_2_ to NH_3_ can produce partial nitridation and lateral phase evolution [48]. More broadly, conversion‐driven reconstruction can generate local strain and rotational disorder near partially converted regions. These features may influence charge and phonon transport as well as interfacial electronic structure, but they should be distinguished from a periodic moiré superlattice unless the corresponding registry modulation is directly resolved. Control over polytypes and polymorphic phase evolution further expands opportunities for strain engineering across hybrid 2D–3D heterointerfaces [52, 53].

## 6 Ex Situ Transfer and the Pathway to Automation

Transfer‐based assembly constructs heterostructures from pre‐grown or exfoliated 2D materials [34]. Wet‐transfer methods remain widely used for large‐area integration and substrate exchange because they are simple and scalable, but they offer limited control over rotational alignment, interfacial cleanliness, and local strain. These limitations are especially important in moiré systems, where electronic behavior depends sensitively on twist angle, including low‐angle graphene structures [22] and 30° graphene homostructures [54]. Deterministic or semi‐dry transfer methods address part of this problem by controlling stacking and twist angle between individual layers. Unlike direct growth, deterministic transfer broadens the accessible material/substrate combinations while enabling systematic studies of angle‐dependent interfaces. Atomically clean interfaces and precise rotational alignment, however, remain difficult to maintain during transfer.

Representative deterministic transfer approaches commonly employ polymer‐assisted handling layers such as polymethyl methacrylate (PMMA) and polydimethylsiloxane (PDMS), combined with rotational transfer stages and micromanipulation systems, as illustrated in Figure 1D,E [34, 35]. These approaches enable controlled assembly of vertical heterostructures across a broad range of material systems and substrates, while providing tunable control over interfacial strain and moiré reconstruction.

Recent advances in transfer‐based assembly have improved control over interface alignment and local lattice reconstruction in heterostructures [22, 55]. Automation and data‐assisted optimization are also beginning to accelerate high‐throughput assembly workflows [30]. Beyond conventional van der Waals assembly, transfer‐based integration now extends to hybrid architectures that combine 2D materials with 3D substrates, flexible supports, or polaritonic systems [29, 30, 31].

## 7 Experimental Characterization of Strained and Twisted Structures

Strain and twist are often spatially nonuniform; therefore, no single characterization method fully captures the coupled structural and electronic response. Multimodal characterization is needed to connect lattice deformation with local stacking, interlayer coupling, and transport behavior. Studies of strain‐engineered 2D materials commonly rely on nano‐ to microscale imaging and spectroscopy that probe lattice deformation and its electronic consequences. In strained systems, deformation is frequently introduced through flexible or patterned substrates, enabling controlled modulation of local strain fields and interfacial coupling.

In strained MoS_2_ photodetectors deposited on flexible substrates, strain‐dependent modulation of *I*–*V* characteristics reveals enhanced piezoelectric response under applied deformation (0.8%–1.4% strain) (Figure 2A). Strain can also be introduced through nanoscale surface texturing, where ridges or nanopillar/hole arrays locally reshape strain distribution within atomically thin layers. In graphene, textured substrates with pitch lengths ranging from 40–250 nm generate measurable shifts in Raman G (∼1582 cm^−1^) and 2D (∼2697 cm^−1^) modes, enabling quantitative mapping of local strain and lattice conformation across patterned architectures (Figure 2B,C) [56, 57].

**FIGURE 2:**
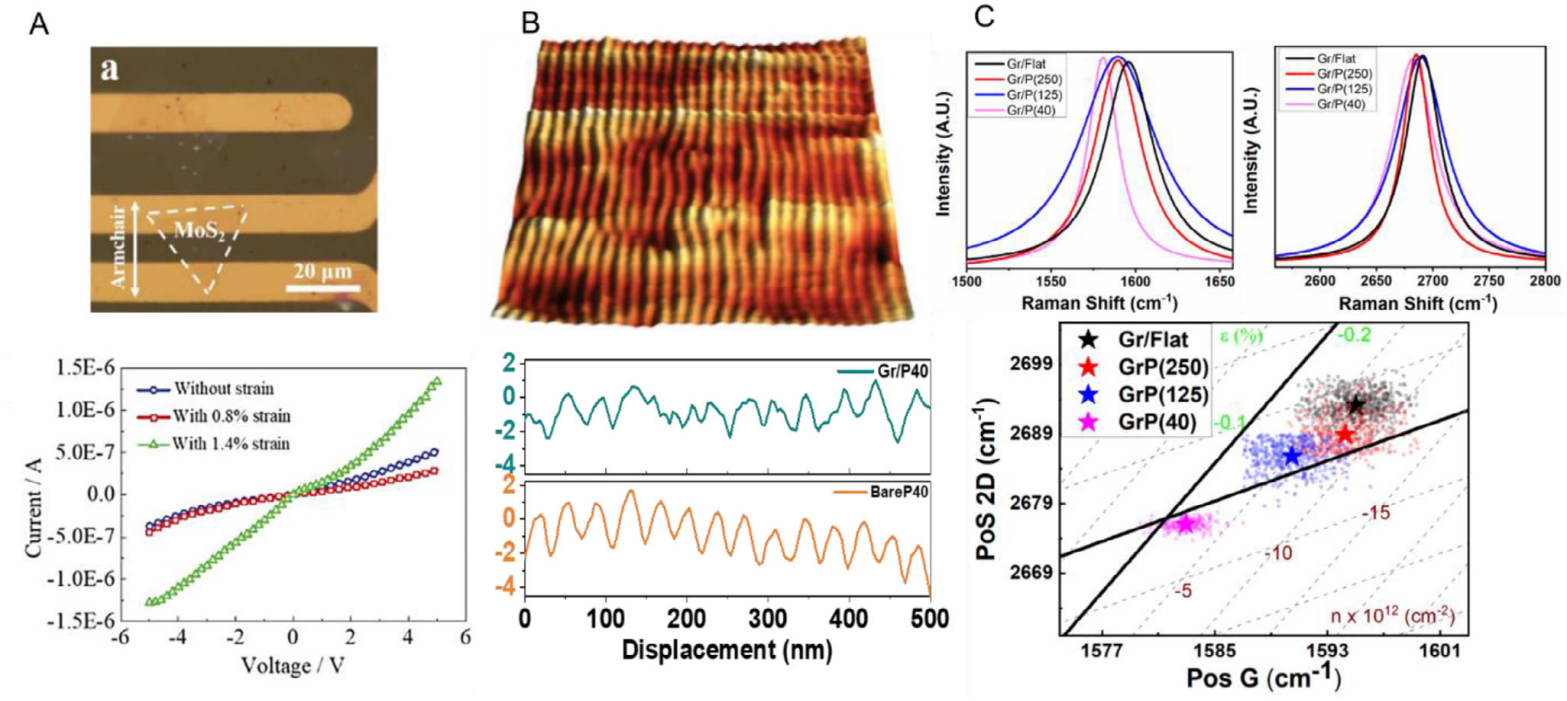
Strain application and multimodal characterization in 2D materials. (A) Optical micrograph of a monolayer MoS_2_ device spanning electrodes and current–voltage characteristics measured under applied strains of 0.8%–1.4%. (B) AFM topography and line profiles of a 40 nm‐pitch textured surface before and after graphene coverage. (C) Raman G‐ and 2D‐mode spectra measured on textures with pitch lengths of 40–250 nm, together with a G–2D correlation plot used to assess the strain response. Adapted from [56, 57].

Direct imaging and reciprocal‐space characterization have been central to resolving the structural complexity of moiré systems. High‐resolution annular dark‐field scanning transmission electron microscopy (ADF‐STEM), scanning tunneling microscopy (STM), nanobeam diffraction, and 4D‐STEM can reveal lattice reconstruction, stacking order, moiré periodicity, and rotational domains, as summarized in Figure 3A,B. At small twist angles, structural relaxation in bilayer graphene expands the low‐energy AB and BA domains, while the higher‐energy AA regions shrink, and concentrate near intersections of the domain‐wall network [43, 58]. Fourier analysis of real‐ or reciprocal‐space data can then be used to quantify local moiré periodicity and strain/rotation heterogeneity.

**FIGURE 3:**
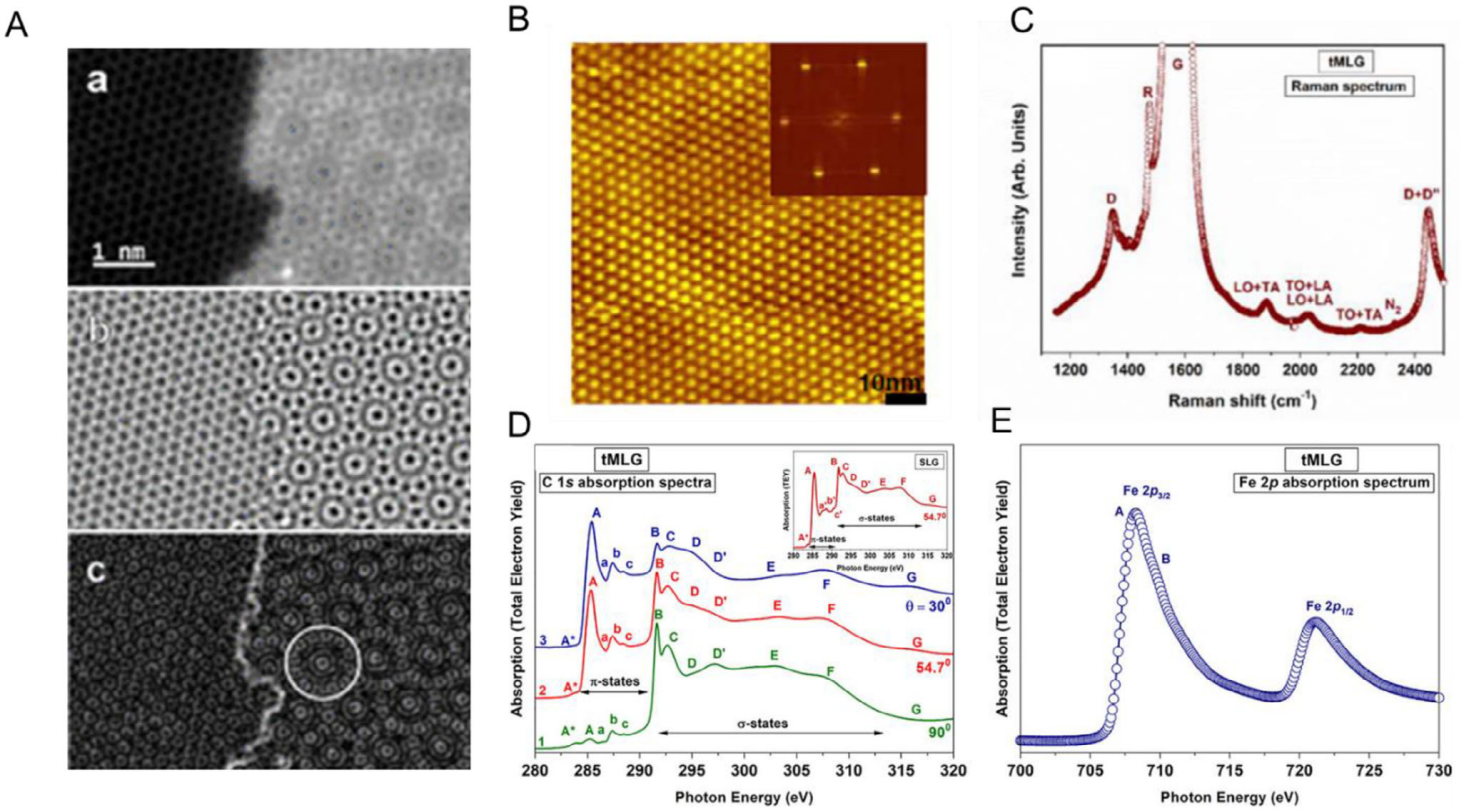
Structural, vibrational, and chemical characterization of twisted graphene. (A) ADF‐STEM image of twisted graphene at a local angle of 28.4°, together with the corresponding phase and Fourier‐magnitude analyses. (B) STM micrograph of a moiré pattern with a twist angle of 3.5 ± 0.3°; the inset Fourier transform contains contributions from the atomic lattice and moiré superstructure. (C) Raman spectrum of twisted multilayer graphene showing the D, G, rotational R, D + D′, and combination modes. (D) C 1s NEXAFS spectra collected at X‐ray incidence angles of 30°, 54.7°, and 90°, with a single‐layer graphene spectrum shown for comparison. (E) Fe 2p spectrum identifying iron‐containing impurities associated with the substrate. Adapted from [43, 58].

Electronic characterization techniques including scanning tunneling spectroscopy (STS), Landau‐level spectroscopy, and density‐of‐states mapping reveal the strong dependence of electronic transport and correlated behavior on twist angle in graphene‐based moiré systems. At large twist angles (>20°), twisted graphene layers exhibit behavior similar to electronically decoupled monolayers, including linear conductance spectra and carrier mobilities ranging from ∼10 000–100 000 cm^2^ V^−1^ s^−1^ at room temperature [44, 58]. As the twist angle decreases, enhanced interlayer coupling progressively modifies carrier velocity and promotes the emergence of van Hove singularities and flat‐band behavior. Near the magic angle (∼1.1°), the energy separation between van Hove singularity peaks decreases to ∼32 meV, consistent with the emergence of strongly correlated electronic phases [59].

Complementary spectroscopic techniques provide additional signatures of twist‐dependent interlayer coupling and structural quality in multilayer graphene systems. Raman spectroscopy reveals changes in the D, G, 2D, and rotational R modes as a function of twist angle, resonance condition, layer number, strain, and disorder (Figure 3C) [44]. The near‐edge X‐ray absorption fine‐structure (NEXAFS) spectra in Figure 3D were collected at different X‐ray incidence angles and probe the orientation and unoccupied electronic states of sp^2^‐bonded carbon. Carbon 1s X‐ray photoelectron spectroscopy (XPS) can quantify sp^2^, sp^3^, and oxidized carbon components. The Fe 2p spectrum in Figure 3E instead probes iron‐containing substrate impurities [43].

## 8 Influence of Strain and Twist in Magnetic Materials

Magnetic 2D materials provide a useful case study because both strain and local stacking directly modify exchange interactions. Magnetic order in two‐dimensional materials is highly sensitive to crystal structure and interlayer registry, including local lattice distortion, allowing strain and twist to tune magnetic behavior without changing composition. Unlike conventional magnetic tuning strategies that rely on composition or external magnetic fields, straintronics, and twistronics can continuously tune magnetic phases through mechanical deformation and rotational alignment. In this context, strain primarily tunes magnetic exchange through local changes in bond length, bond angle, and orbital overlap, whereas twist introduces spatially periodic variations in stacking‐dependent interlayer coupling.

Early studies have established that magnetic order in 2D materials is strongly coupled to structure. In CrI_3_, pressure‐ or stacking‐induced structural changes can switch interlayer coupling between ferromagnetic (FM) and antiferromagnetic states [60, 61]. In twisted TMD systems such as MoSe_2_, the interplay between moiré reconstruction and strain can generate soliton networks that confine charge carriers and create tunable quantum channels [62]. More broadly, strain can drive magnetic transitions by modifying exchange interactions and spin alignment [63].

## 9 Strain‐Induced Magnetic Phase Control

Strain directly modifies magnetic exchange interactions through changes in bond lengths, bond angles, and orbital overlap [63]. This provides a route for controlling magnetic anisotropy, coercivity, and magnetic phase stability without altering chemical composition. A representative example is the van der Waals ferromagnetic metal Fe_3_GeTe_2_ (FGT), where tensile strain is applied using a three‐point bending geometry, as illustrated in Figure 4A [64]. Hall‐effect measurements reveal a strong correlation between coercive field and applied strain, leading to strain‐driven magnetization reversal. These observations demonstrate how relatively modest lattice distortions can directly alter magnetic switching behavior.

**FIGURE 4:**
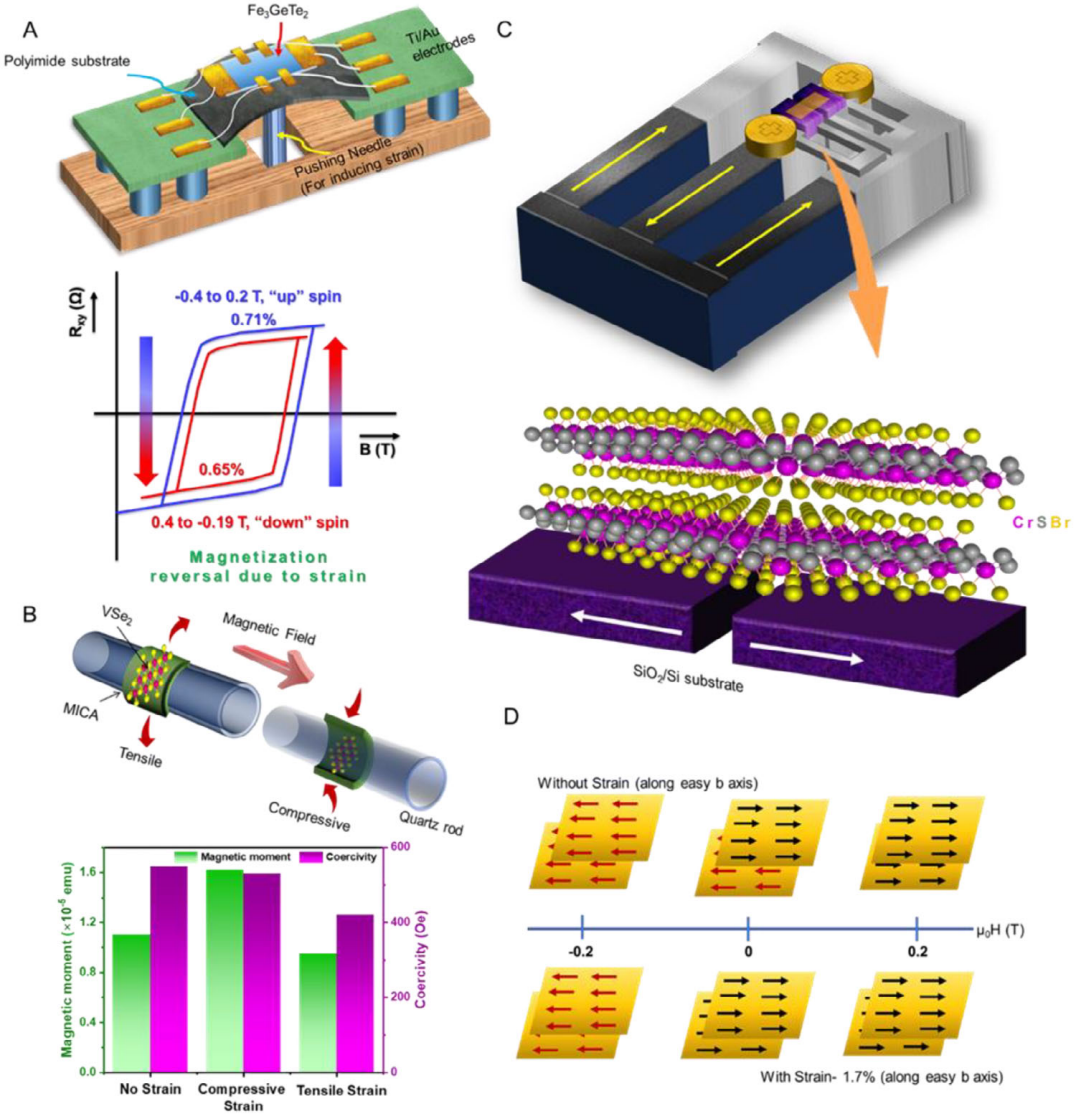
Strain‐induced changes in 2D magnetic materials. (A) Three‐point bending geometry for Fe_3_GeTe_2_ and representative Hall hysteresis loops showing reversal between up and down magnetization states as tensile strain changes from 0.71% to 0.65%. (B) Tensile and compressive deformation of VSe_2_ on a flexible support, with the corresponding magnetic moment and coercivity. (C) Cryogenic strain cell comprising three piezoelectric stacks and a suspended CrSBr flake spanning a micrometer‐scale gap in a SiO_2_/Si substrate. (D) Schematic CrSBr magnetic states without strain and under approximately 1.7% strain applied along the crystallographic *b* axis. Adapted from [64, 65, 66].

Strain‐controlled magnetic tuning has also been demonstrated in VSe_2_ nanoflakes grown on flexible substrates (Figure 4B) [65]. Tensile strain increases the saturation magnetization from approximately 1.1 × 10^−5^ emu to 1.62 × 10^−5^ emu, whereas compressive strain reduces it to approximately 9.5 × 10^−6^ emu. These changes are attributed to strain‐induced modifications in atomic spacing and magnetic exchange interactions. Recent advances have extended strain engineering into cryogenic regimes through specialized strain‐cell architectures. As shown in Figure 4C, micrometer‐scale suspended CrSBr flakes can be strained across a controlled substrate gap, enabling large local deformations without damaging the material [66]. Under approximately 1.7% strain, CrSBr undergoes a spin‐flip transition into a field‐induced ferromagnetic state (Figure 4D), highlighting the ability of strain to directly manipulate magnetic ground states.

Several layered magnetic systems also exhibit strain‐dependent magnetic phase transitions. TMD‐based magnetic materials such as NbS_2_ and NbSe_2_, along with chromium trihalides (CrX_3_; X = I, Br, and Cl), display magnetic ordering that depends strongly on lattice geometry and stacking configuration [67]. In CrI_3_, magnetic order remains stable up to approximately 2% tensile strain, beyond which the material undergoes a transition from a magnetic metal to a half‐metal and eventually to an antiferromagnetic half‐semiconductor state [68]. These transitions originate from strain‐induced changes in hybridization between Cr d‐orbitals and halogen p‐orbitals.

## 10 Twisted Magnets and Moiré Magnetic Textures

Twisted van der Waals magnets provide a complementary route for controlling magnetism through spatial modulation of interlayer exchange interactions [69]. As the twist angle varies, the resulting moiré superlattice generates local stacking environments with distinct magnetic ground states, producing complex magnetic domain structures and emergent spin textures. Bilayer CrI_3_ has emerged as a model system for studying moiré magnetism. Scanning magnetometry based on nitrogen‐vacancy centers, shown schematically in Figure 5A, enables direct imaging of local magnetic fields within twisted structures [70]. The resulting magnetic landscape is governed by local stacking geometry. As illustrated in Figure 5B,C, rhombohedral stacking favors ferromagnetic (FM) coupling, whereas monoclinic stacking favors antiferromagnetic (AFM) coupling. Small twist angles therefore generate periodic FM and antiferromagnetic domains that follow the moiré superlattice.

**FIGURE 5:**
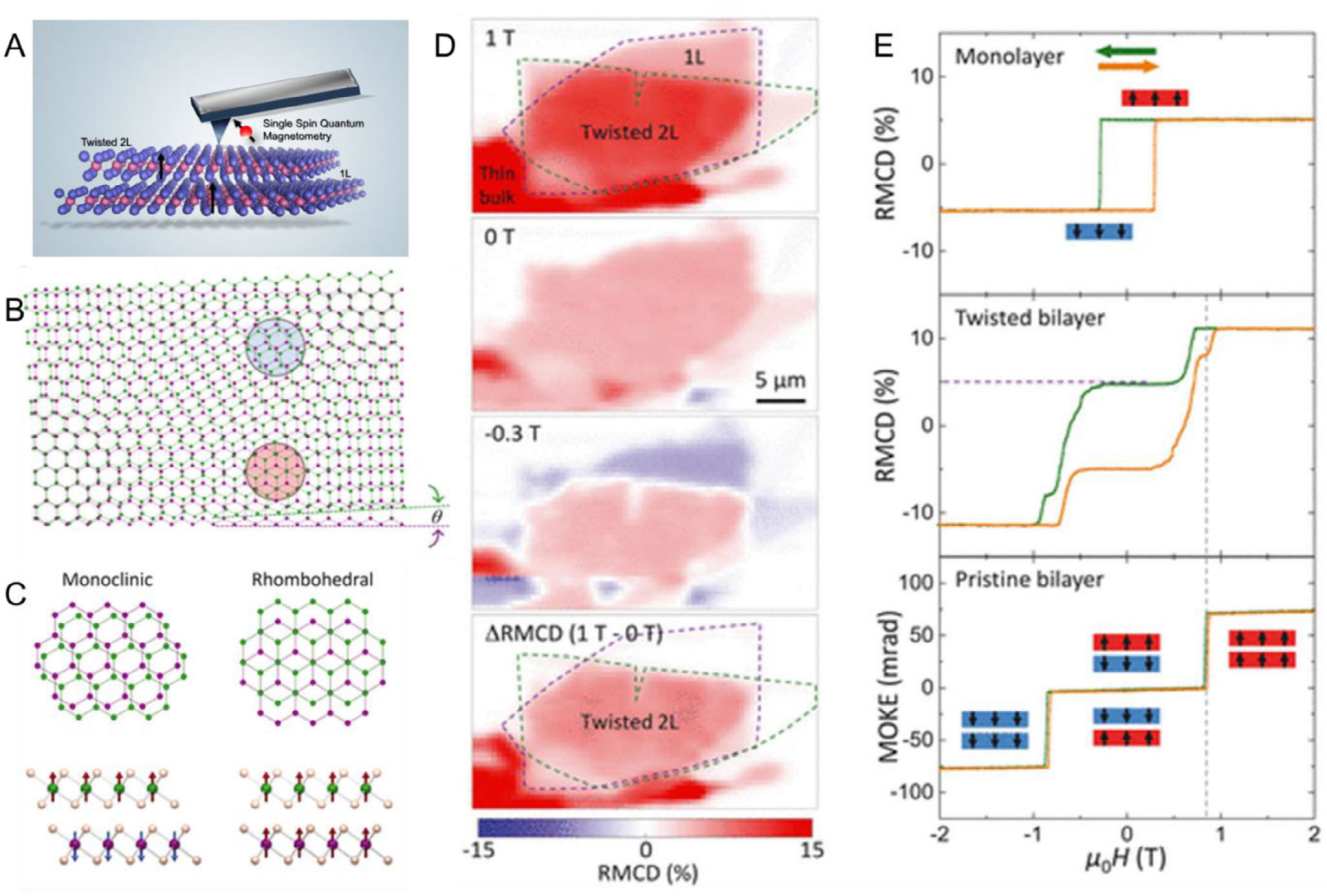
Stacking‐dependent magnetism in twisted bilayer CrI_3_. (A) NV‐center magnetometry integrated with an AFM probe for mapping the local stray magnetic field. (B) Twisted bilayer geometry containing local monoclinic and rhombohedral stacking regions. (C) Monoclinic and rhombohedral registries associated with antiferromagnetic and ferromagnetic interlayer coupling, respectively. (D) Reflective magnetic circular dichroism maps acquired at µ_0_H = 1, 0, and −0.3 T, together with the difference between the 1 and 0 T maps. (E) RMCD and magneto‐optical Kerr‐effect hysteresis signals for monolayer, pristine bilayer, and twisted bilayer CrI_3_, with the corresponding magnetic configurations shown in the insets.

Magneto‐optical techniques such as reflective magnetic circular dichroism (RMCD) and magneto‐optical Kerr effect (MOKE) provide complementary probes of these twist‐dependent magnetic textures. Figure 5D presents RMCD maps acquired as the magnetic field is varied, revealing the spatial evolution of magnetic domains within twisted bilayer CrI_3_, while Figure 5E compares RMCD and MOKE hysteresis loops for monolayer, pristine bilayer, and twisted bilayer structures. These measurements directly demonstrate how rotational alignment modifies magnetic ordering. Beyond simple FM and AFM ordering, twisted magnets can host non‐collinear spin textures and topological magnetic excitations. The coexistence of stacking‐dependent interlayer exchange and Ising‐like intralayer ferromagnetism can generate magnetic domain walls that support localized spin‐wave modes and topological magnon excitations [71]. Such states provide a platform for exploring low‐power spin transport and non‐volatile information processing architectures.

## 11 Coupled Electronic, Magnetic, and Lattice Effects

The influence of strain and twist extends beyond magnetic ordering alone because moiré systems often couple lattice relaxation with electronic reconstruction and collective excitations. Although not a layered magnet, magic‐angle twisted bilayer graphene (MATBG) illustrates how these degrees of freedom can become intertwined. In MATBG, angle‐resolved photoemission spectroscopy (ARPES) and atomic force microscopy have revealed flat‐band replicas separated by approximately 150 meV, attributed to strong intervalley electron–phonon coupling involving graphene transverse optical phonons [72, 73]. These observations show that lattice vibrations can substantially reshape the electronic landscape of correlated moiré systems. Translating strain‐ and twist‐engineered magnetism into spintronic or quantum‐information technologies will require better control of twist‐angle homogeneity and strain/defect distributions, together with characterization tools that resolve magnetic textures across multiple length scales [74].

## 12 Strain and Twist in Photovoltaic Materials and Halide Perovskites

Halide perovskites provide a complementary optoelectronic case study because their soft lattices make their electronic and excitonic states particularly responsive to structural distortion. Strained and twisted 2D materials are relevant to photovoltaic and optoelectronic technologies because light absorption and exciton‐mediated charge separation are sensitive to lattice deformation and interlayer coupling. TMDs such as MoS_2_, WS_2_, and WSe_2_ have shown strain‐tunable band gaps and valley‐selective photocurrent responses, while twisted TMD bilayers introduce moiré excitons and flat‐band phenomena with potential relevance to excitonic solar‐energy conversion [75, 76, 77, 78]. Layered two‐dimensional halide perovskites (2D HaPs) are especially attractive in this context because their soft lattices and strong excitonic character make them highly responsive to both strain‐induced lattice distortion and twist‐induced interlayer reconstruction.

Structurally, 2D HaPs consist of atomically thin inorganic perovskite layers separated by organic spacer molecules, forming natural quantum‐well architectures with strong dielectric confinement [79, 80, 81]. Their optoelectronic properties can be tuned through layer thickness/composition and interlayer interactions, leading to large exciton binding energies, tunable band gaps, and strong spin–orbit coupling [82, 83, 84]. These characteristics make 2D HaPs particularly sensitive to both strain‐induced lattice distortions and twist‐induced interlayer reconstruction.

## 13 Engineering Strain in Layered and Three‐Dimensional Halide Perovskites

Strain engineering in 2D HaPs primarily operates through distortion of the inorganic octahedral framework, allowing band structure, carrier transport, excitonic behavior, and phase stability to be modified [85, 86, 87]. Both externally applied strain and internally generated strain from lattice or thermal‐expansion mismatch can produce measurable changes in photovoltaic performance [88, 89]. As shown in Figure 6A, nanopillar‐induced deformation of (BA)_2_(MA)_n−1_Pb_n_I_3n+1_ microplates produces composition‐dependent band gap shifts, with reported magnitudes of approximately 5.60 meV per percentage point of strain through distortion of the Pb─I octahedral framework [85]. Strain‐induced octahedral tilting in layered perovskites such as (C_6_H_5_CH_2_CH_2_NH_3_)_2_Cs_3_Pb_4_Br_13_ similarly transforms a single photoluminescence peak into multiple spectrally resolved features under biaxial strain (Figure 6B), illustrating the sensitivity of excitonic states to lattice deformation [86]. Beyond optical modulation, theoretical studies predict power conversion efficiencies approaching 23.8% in strained low‐dimensional hybrid perovskites under 5% tensile strain [87].

**FIGURE 6:**
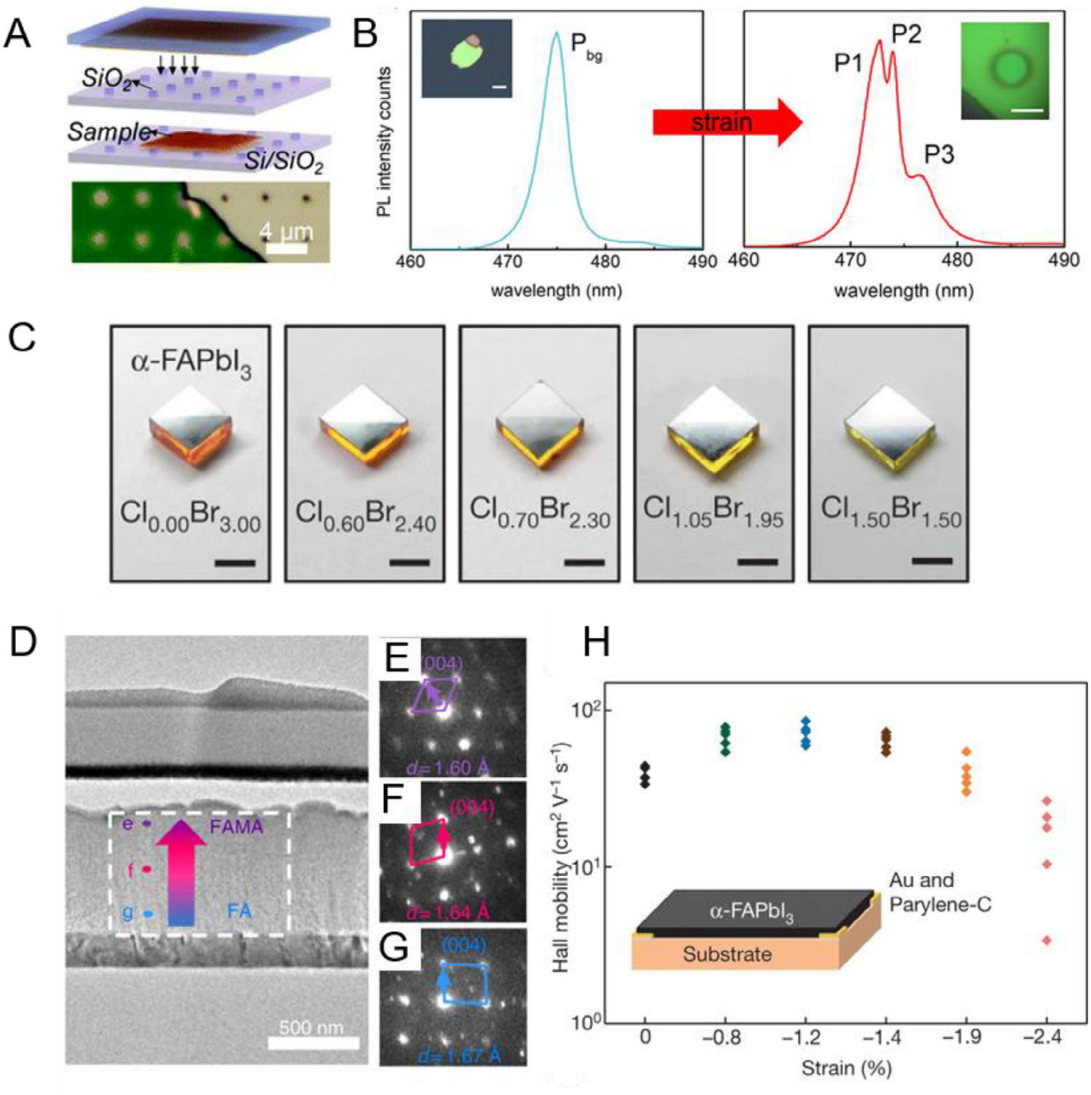
Strain effects in layered and three‐dimensional halide perovskites. (A) Fabrication scheme and optical image of an exfoliated (BA)_2_PbI_4_ microplate deformed over SiO_2_ nanopillars; scale bar, 4 µm. (B) Photoluminescence spectra of a layered perovskite before and after biaxial deformation on a SiO_2_ ring; scale bars, 20 µm for the unstrained flake and 10 µm for the strained region. (C) Optical images of epitaxial α‐FAPbI_3_ films grown on substrates with different lattice mismatch; scale bar, 4 mm. (D) Cross‐sectional TEM image of the perovskite device. (E–G) Nanobeam electron‐diffraction patterns acquired at the positions marked in panel D, revealing a depth‐dependent strain distribution. (H) Hall mobility of α‐FAPbI_3_ as a function of compressive strain. Adapted from [85, 86, 88, 89].

Strain also plays an important role in controlling phase stability and charge transport in halide perovskites beyond the strictly layered 2D family. Compressive strain generated through lattice mismatch during epitaxial growth of three‐dimensional α‐FAPbI_3_ on MAPbCl_x_Br_3−x_ substrates reaches values as high as 2.4%, reduces the band gap by approximately 35 meV, increases hole mobility, and stabilizes the α phase for at least 360 days after growth (Figure 6C,H) [88]. Depth‐dependent diffraction measurements further reveal residual strain gradients in mixed perovskites, where tensile strain near the surface and compressive strain near the substrate influence band bending, carrier extraction, and recombination dynamics (Figure 6D–G) [89]. Engineering these distributions has produced power‐conversion efficiencies as high as 20.7% in the cited device system. Together, these three‐dimensional examples show that strain‐mediated phase stabilization and transport extend beyond the layered halide‐perovskite family.

## 14 Moiré Superlattices in Ultrathin Halide Perovskites

Theoretical studies first predicted that twisted 2D halide perovskites could host flat bands and moiré excitons analogous to those observed in twisted TMDs and graphene systems [90]. Compared with conventional van der Waals heterostructures, interlayer coupling in 2D HaPs may be enhanced through the combined effects of van der Waals interactions, hydrogen bonding, and deep moiré potentials [91, 92, 93]. Realizing these phenomena experimentally requires atomically flat, ultrathin, and ligand‐free perovskite layers, since bulky organic ligands can suppress electronic coupling between adjacent inorganic sheets [91, 92, 93].

A major experimental breakthrough was reported by Zhang et al., who developed ligand‐exchange strategies for producing ultrathin ligand‐free APbX_3_ (A = MA or FA; X = Br or I) nanosheets suitable for deterministic twisting and moiré assembly (Figure 7A,B) [94, 95]. Twisted structures fabricated with angles between approximately 8° and 12° exhibit localized moiré excitons, enhanced photoluminescence exceeding an order of magnitude, reduced exciton diffusivity, and suppressed non‐radiative recombination (Figure 7C,D) [94]. Related studies demonstrated deterministic assembly of ultrathin MAPbI_3_ nanosheets with controlled twist angles down to ∼3.2°, revealing localized moiré effects and symmetry‐driven electronic reconstruction through an equilibrium solution growth strategy (Figure 7E) [95]. These advances establish twisted 2D perovskites as a setting for investigating excitonic moiré physics and quantum optoelectronics [96].

**FIGURE 7:**
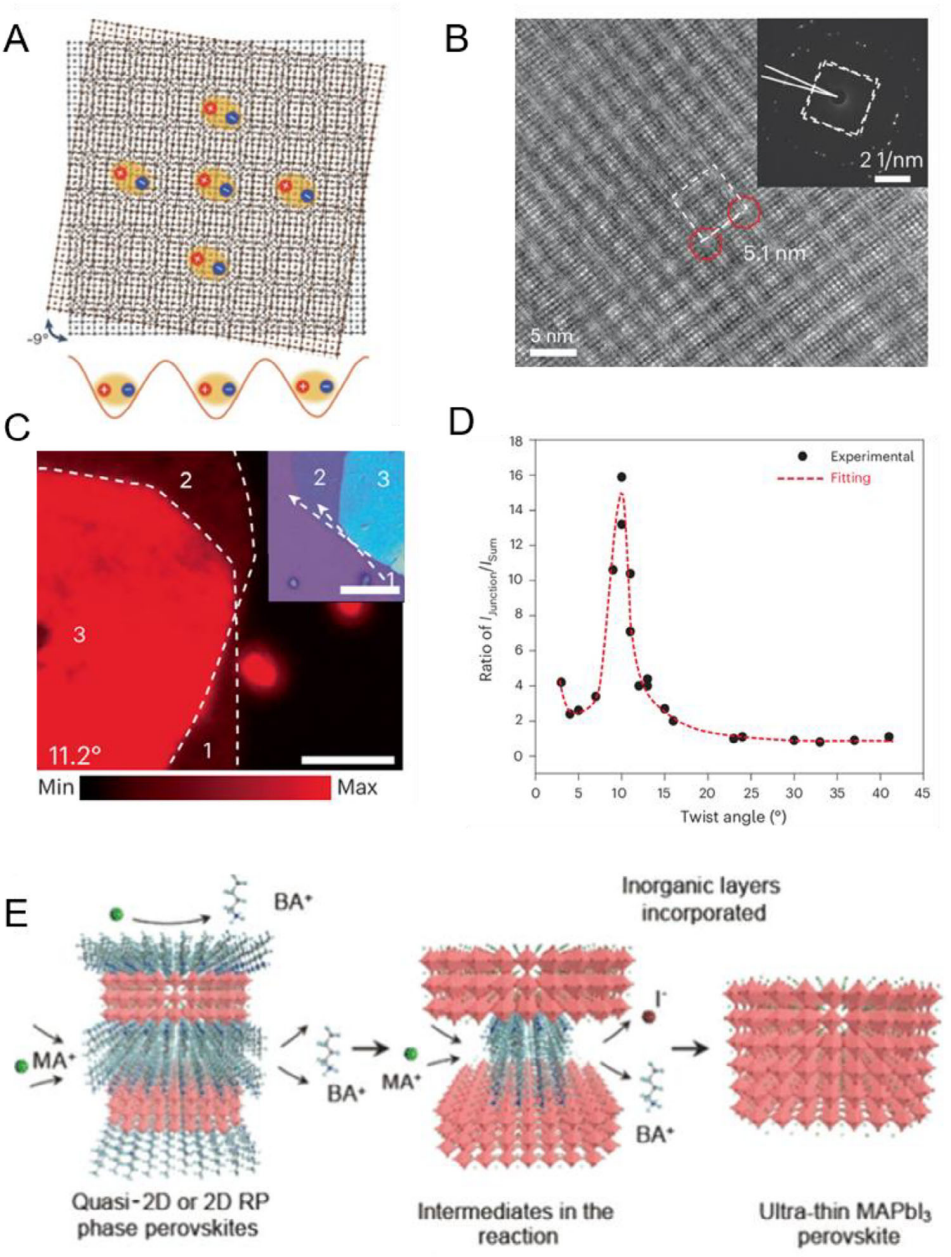
Moiré optical response in ultrathin halide‐perovskite layers. (A) Conceptual illustration of exciton localization in the periodic potential of a twisted perovskite bilayer. (B) High‐resolution transmission electron microscopy image of a 15°‐twisted MAPbBr_3_ bilayer, with the moiré periodicity indicated in the Fourier analysis. (C) Spatially resolved confocal photoluminescence map of an 11.2° MAPbI_3_ twisted structure; scale bar, 10 µm. (D) Angle‐dependent photoluminescence enhancement, expressed as the intensity at the overlap region relative to the sum of the two isolated layers. (E) MAI/PbI_2_ equilibrium‐solution route used to convert quasi‐2D precursor structures into ultrathin MAPbI_3_ layers for deterministic assembly. Adapted from [94, 95, 96].

Beyond standalone 2D HaPs, heterostructures with other 2D materials provide additional opportunities for engineering charge transfer and photovoltaic performance. Type‐II band alignment in perovskite–WS_2_ heterostructures promotes efficient exciton separation and enhanced photoluminescence [93], while vertically stacked perovskite–MoS_2_ architectures exhibit ultrafast interfacial charge transfer on femtosecond timescales [97]. MoS_2_‐based transport layers have also improved charge extraction and operational stability in perovskite solar cells [98], and wafer‐scale monolayer MoS_2_ offers a pathway toward scalable device integration [99]. The strong interfacial coupling and tunable band alignment in these systems could support strain‐ and twist‐engineered photovoltaic architectures. Key practical barriers remain twist‐angle control, strain uniformity, interface quality, and environmental stability. While MAPbI_3_ enabled many of the initial demonstrations, more robust inorganic perovskites may provide a pathway toward practical devices. Deterministic assembly and scalable strain‐engineering strategies will be important for translating moiré and strain phenomena into photovoltaic and optoelectronic architectures.

## 15 Theory and Computational Modeling

Modeling strained and twisted 2D materials is challenging because the relevant physics extends from local bonding to device‐scale deformation (Figure 8). Moiré superlattices are not rigid, so mechanical relaxation is essential to a quantitative description of twisted van der Waals systems. At small twist angles, competition between intralayer elastic energy and interlayer adhesion drives spontaneous lattice reconstruction. The moiré pattern can relax into large commensurate domains, such as alternating AB and BA regions in bilayer graphene, separated by narrow solitonic domain walls [100, 101, 102]. Significant in‐plane strain fields and out‐of‐plane corrugation accompany this reconstruction [103, 104, 105, 106]. Continuum elasticity models describe interlayer coupling through generalized stacking‐fault energy functionals [100, 104, 106]. Related formulations and extensions are reported elsewhere [107, 108]. Atomistic simulations use registry‐dependent interlayer potentials, while multiscale schemes bridge atomistic and continuum descriptions [103, 109, 110]. The resulting deformation fields can generate pseudomagnetic fields, reshape electronic flat bands, and modify phonon spectra and ultralow‐frequency Raman signatures. Predictive treatment must therefore place the mechanical relaxation problem and the electronic or vibrational calculation on equal footing.

**FIGURE 8:**
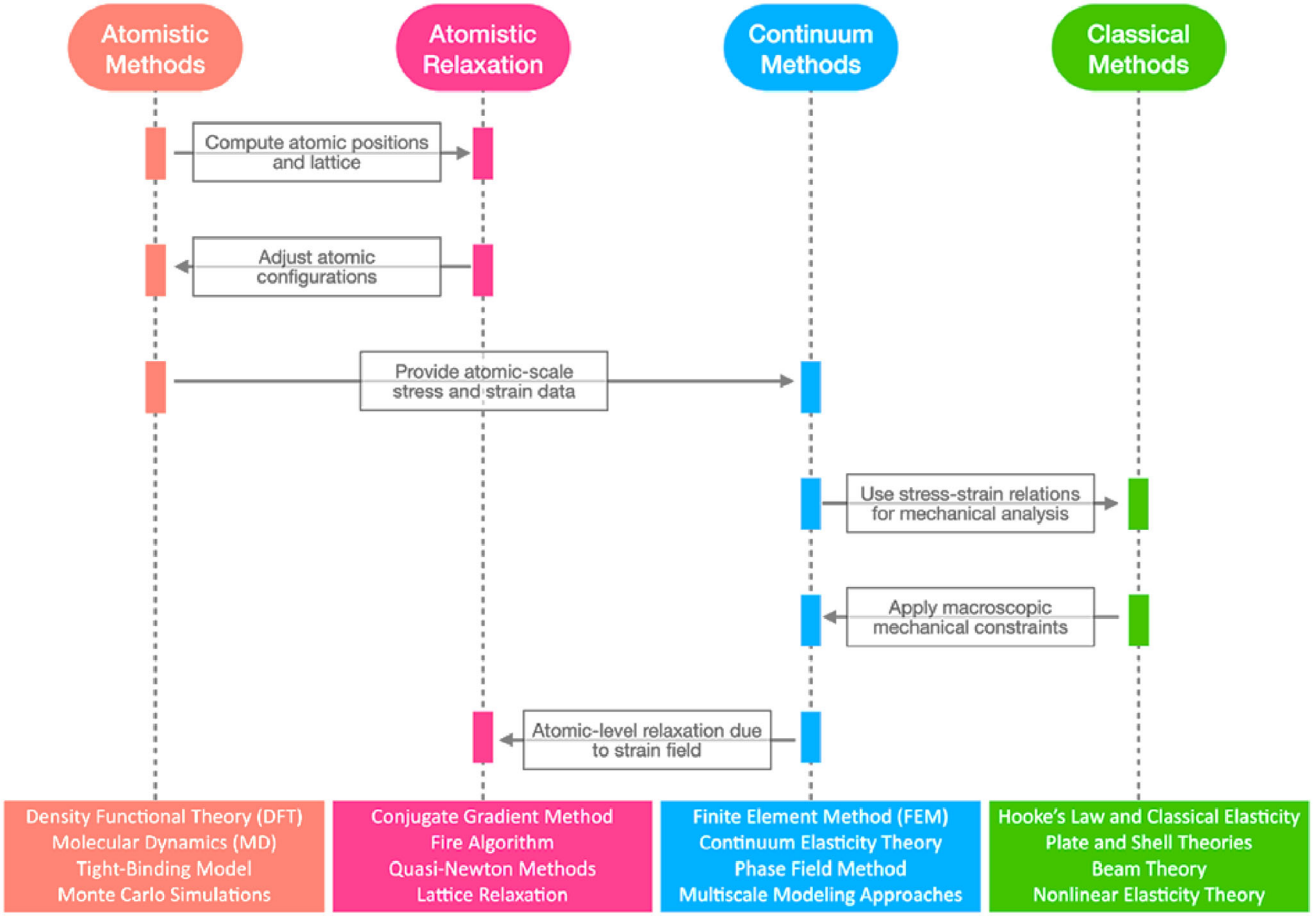
Multiscale modeling workflow for straintronics and twistronics. First‐principles calculations provide local bonding, stacking‐energy, and electronic‐structure information. Registry‐dependent atomistic or machine‐learned potentials extend structural relaxation to large moiré supercells. Continuum elasticity describes long‐wavelength reconstruction, corrugation, and device boundary conditions. The relaxed geometry then enters electronic, excitonic, magnetic, phonon, and transport models, while experimental measurements calibrate and validate each level.

DFT captures local chemistry and strain‐dependent electronic structure, while tight‐binding and continuum models extend electronic‐structure predictions to large moiré supercells. Molecular dynamics, continuum elasticity, and finite‐element approaches connect atomic relaxation to mesoscopic strain redistribution and device‐relevant geometries. At the atomic scale, DFT and related atomistic models show how local distortions modify electronic structure, interlayer hybridization, and chemical bonding [111]. At larger length scales, continuum approaches efficiently describe moiré superlattices, strain fields, and long‐wavelength reconstruction that extend beyond the reach of conventional first‐principles supercells [112]. The Bistritzer–MacDonald continuum model is a landmark example as it predicts magic‐angle behavior in twisted bilayer graphene while remaining computationally tractable for experimentally relevant systems [113].

The strengths of these methods are most apparent when they are combined. Structural relaxation is often resolved first with atomistic calculations that capture local bond distortions and stacking‐dependent reconstruction. These results can then inform continuum elasticity frameworks that describe deformation across experimentally relevant dimensions [100, 102]. Continuum electronic models capture the dominant features of band‐structure evolution, while atomistic calculations provide corrections from local chemistry and correlation effects that are inaccessible to simplified descriptions [114]. Ab initio‐derived continuum models use DFT calculations to parameterize effective continuum Hamiltonians for large‐scale moiré systems [111, 114].

Such multiscale approaches have been particularly important in twistronics, where small changes in twist angle can dramatically alter electronic behavior. Combined theoretical frameworks facilitate the elucidation of the emergence of moiré superlattices, flat bands, and correlated electronic phases in twisted 2D materials [113]. In straintronics, atomistic simulations reveal how local strain modifies bonding and electronic structure, while continuum descriptions connect these effects to device‐scale behavior [63, 115, 116]. As a result, theoretical modeling now spans length scales ranging from atomic reconstruction to complete device architectures through combinations of DFT, tight‐binding methods, molecular dynamics, continuum elasticity, and finite‐element modeling.

The growing convergence of straintronics and twistronics has further increased the importance of multiscale theory. Recent moiré band‐theory studies show that strain‐induced lattice reconstruction can modify van Hove singularities and flat‐band formation in twisted systems [117]. These advances are increasingly complemented by machine learning approaches that accelerate exploration of large parameter spaces and connect atomistic simulations to device‐level models.

A remaining challenge is to bridge atomic‐scale physics with experimentally relevant device dimensions. Meeting this challenge will be essential for coupled strain‐twist physics and predictive modeling of electronic and quantum technologies [118].

## 16 Machine Learning for Straintronics and Twistronics in 2D Materials

Machine learning (ML) is becoming increasingly useful in straintronics and twistronics, where subtle strain gradients and twist‐angle‐dependent interlayer coupling can exceed conventional analysis workflows, especially in large experimental datasets. Atomic‐resolution microscopy and electron diffraction provide rich structural information, but their interpretation is limited by noise, beam‐induced damage, and multidimensional data [119, 120, 121, 122, 123]. First‐principles calculations are essential for electronic‐structure analysis but become computationally demanding for large moiré supercells. ML can accelerate characterization and property prediction by automating feature extraction across experimental and computational datasets [124, 125, 126, 127]. Additional studies have extended these methods to real‐time analysis and uncertainty estimation [128, 129, 130]. Related work has considered materials screening and transferable analysis frameworks [131, 132].

## 17 Deep Learning and Neural Networks for Strain Mapping and Analysis

Deep learning methods have improved the extraction of strain fields from microscopy and diffraction datasets in 2D materials. These advances have been driven by neural network architectures that can identify local structural distortions while reducing the dose or acquisition time required for beam‐sensitive materials.

One representative example is the use of a fully convolutional network (FCN) with a ResUNet architecture to analyze atomic‐scale disorder in WSe_2−2x_Te_2x_ (Figure 9A) [121]. This approach combines automated defect recognition with image processing and achieved approximately 0.2 pm precision in the inferred atomic positions used to construct strain maps: a 20‐fold improvement over traditional methods. The system has efficiently analyzed approximately 130 000 atoms across 4000 nm^2^, enabling the first direct observation of complex, oscillating strain fields around atomic defects while minimizing electron beam exposure. This methodology is particularly beneficial for beam‐sensitive 2D materials, where high‐dose measurements often result in significant structural damage.

**FIGURE 9:**
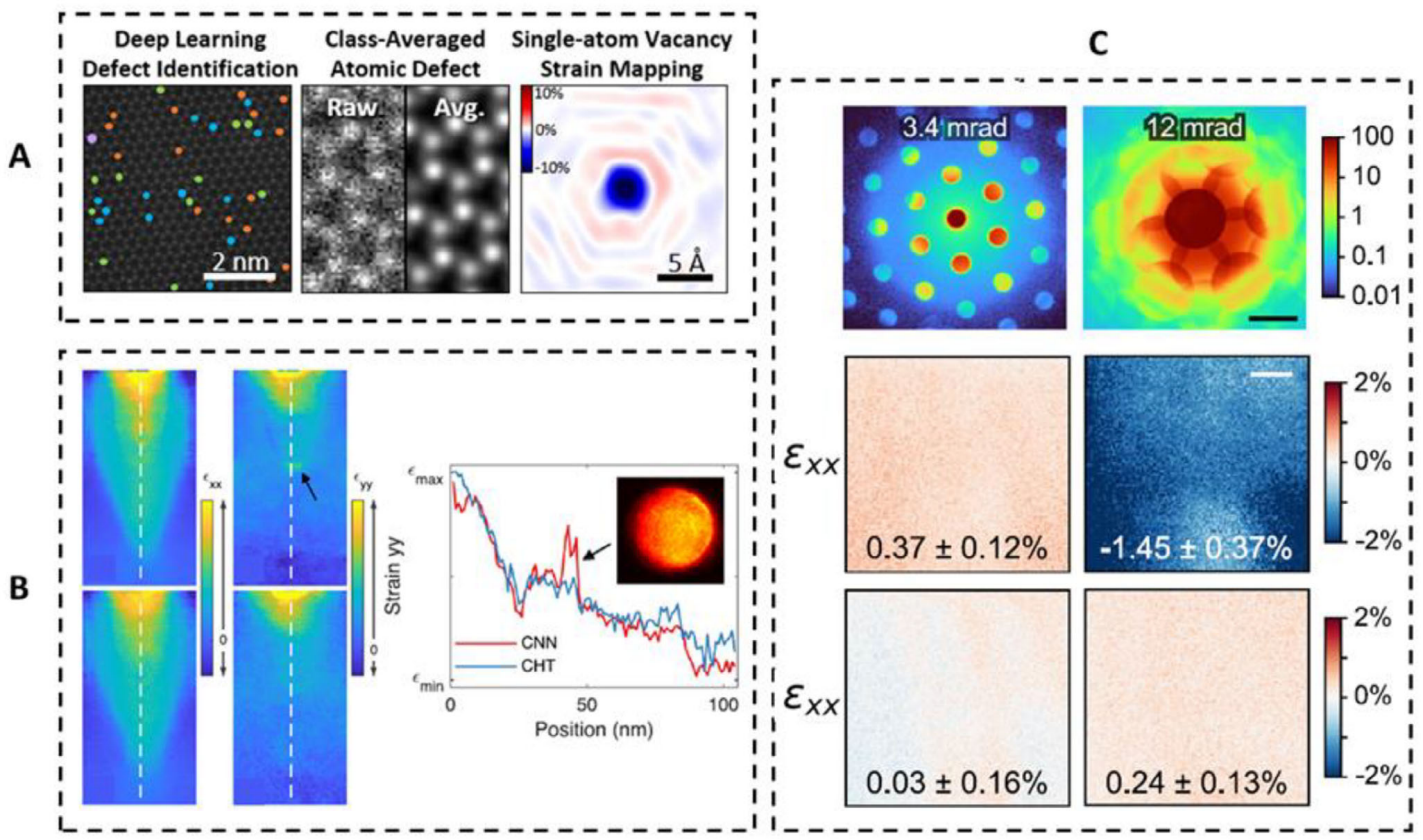
Supervised machine‐learning approaches for strain mapping. (A) Deep‐learning‐based defect identification in WSe_2−2x_Te_2x_, class‐averaged atomic‐resolution images, and a displacement‐derived strain map; the reported approximately 0.2 pm value refers to the precision of the inferred atomic positions. (B) Neural‐network‐assisted diffraction analysis shown as a transferable metrology example. (C) FCU‐Net analysis of h‐BN diffraction patterns acquired with 3.4 and 12 mrad probe semi‐angles, compared with a conventional analysis under increasingly complex multiple‐scattering conditions. Adapted from [121, 122, 123].

Extending ML to diffraction analysis, artificial neural networks, and convolutional neural networks trained on simulated electron diffraction patterns have also been used to automate feature recognition (Figure 9B) [122]. Although this demonstration is not specific to 2D materials, the strategy is relevant to diffraction‐based strain mapping because it targets local diffraction features rather than requiring manual interpretation of full patterns.

Further advancing the field, the Fourier‐space complex U‐Net (FCU‐Net) was developed to address multiple scattering effects in diffraction (Figure 9C) [123]. This model, trained on over 200 000 unique simulated diffraction patterns with realistic experimental conditions, demonstrates high accuracy and robustness. Applied to hexagonal boron nitride (h‐BN), the method outperforms conventional approaches, particularly under challenging conditions such as wide probe angles and overlapping diffraction patterns. The FCU‐Net reduces systematic errors (±0.13% compared to ±0.37% for traditional methods) and maintains accuracy across diverse experimental scenarios, including conditions outside the training set.

Unlike molecular dynamics or finite‐element analysis, these ML methods operate directly on experimental data and can connect measurement with model development. Interpretability remains a central limitation: while ML models excel at pattern recognition, extracting physical meaning from their internal representations requires further development. Current models also face generalization barriers when transitioning between material systems, often requiring extensive retraining because datasets remain biased toward well‐characterized materials. Physics‐informed network architectures may help by incorporating known constraints and making extracted strain features more transparent [133].

These developments in neural‐network analysis have improved strain mapping in 2D materials and are increasingly available through user‐accessible frameworks such as py4DSTEM [134]. Supervised approaches can provide high precision across defined experimental conditions, but their reliance on labeled training data limits their ability to discover unexpected structures. This limitation has increased interest in unsupervised methods that identify recurring patterns without predefined labels.

## 18 Unsupervised Learning for Structural Classification

Supervised learning can be precise, but its reliance on labeled data limits its ability to uncover unexpected structures. Unsupervised machine learning addresses this gap by identifying structural patterns in 4D scanning transmission electron microscopy (4D‐STEM) data without predefined labels [135].

One useful example is divisive hierarchical clustering for analyzing complex heterostructures (Figure 10A) [119]. This method iteratively partitions 4D‐STEM data into finer subgroups, first distinguishing material composition, and then resolving subtler strain‐induced ripples. In WS_2–_WSe_2_ lateral heterojunctions [119], where traditional ADF‐STEM imaging struggles to differentiate regions because tungsten dominates the contrast, hierarchical clustering reveals structural information across multiple scales. The method achieves 99.9% accuracy in distinguishing WS_2_ and WSe_2_ while also identifying rotational variations and ripple patterns that accommodate lattice mismatch strain. These ripples are directly linked to functional properties because they modify local band structures and influence optical behavior.

**FIGURE 10:**
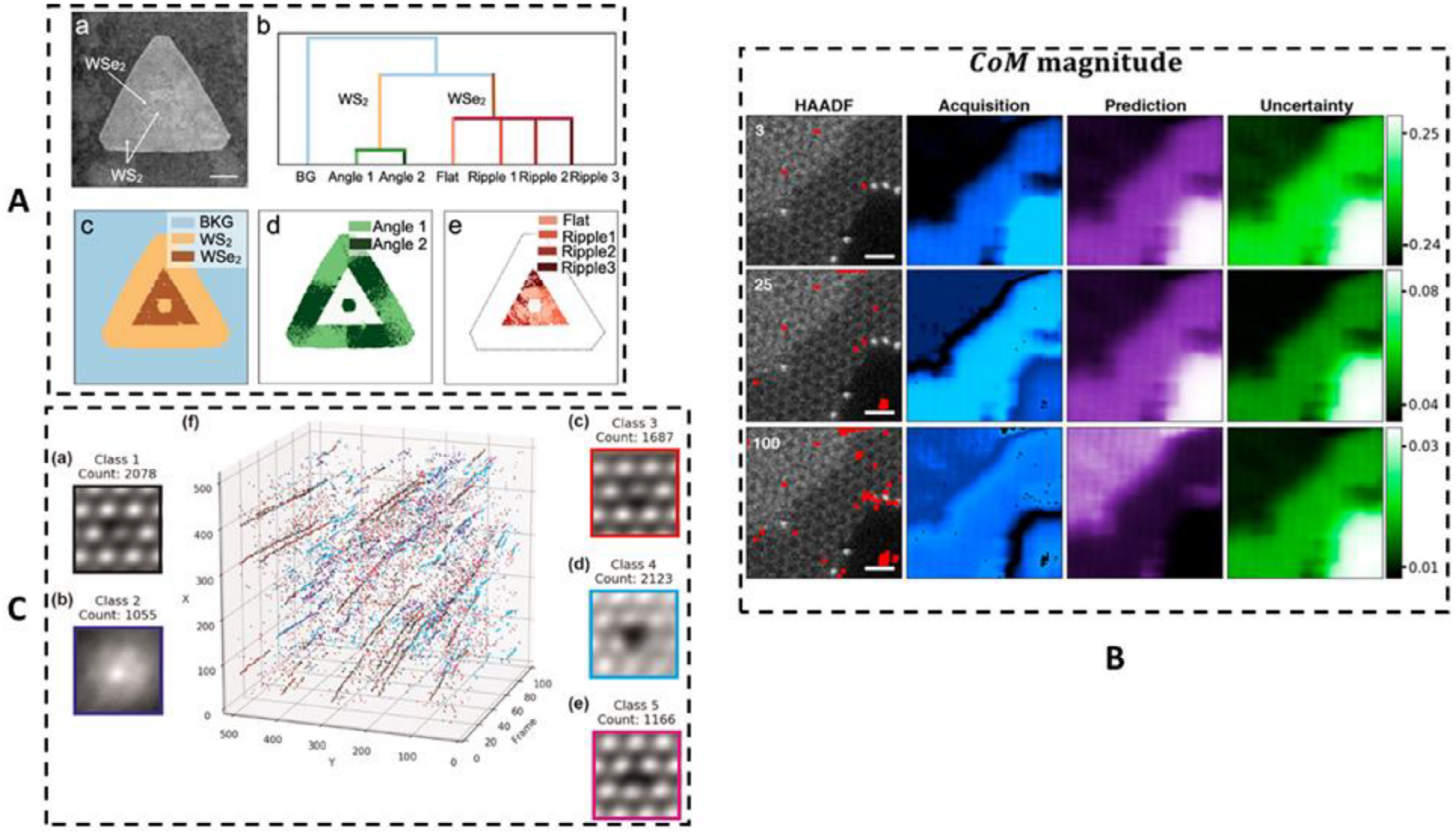
Unsupervised and active‐learning approaches for structural analysis. (A) Divisive hierarchical clustering of a WS_2–_WSe_2_ lateral heterojunction, progressively separating composition, crystal‐orientation variation, and strain‐accommodating ripple patterns. (B) DKL‐guided 4D‐STEM acquisition after 3, 25, and 100 measurements, showing the selected positions, acquisition function, predicted response, and uncertainty. (C) Gaussian‐mixture classification of five defect classes in electron‐irradiated WS_2_ and the corresponding three‐dimensional spatiotemporal defect trajectories. Adapted from [119, 120, 136].

To address complex systems, complementary approaches have been developed for twisted bilayer structures. Using variational Bayesian Gaussian mixture (VBGM) modeling, stacking configurations in bilayer MoS_2_ were analyzed automatically [137]. VBGM automates the identification of stacking domains without manual thresholding. This method eliminates the need for prior knowledge of layer orientations, enabling the detection of domain shapes, stacking orders (AA′, AB, and AC), grain orientations, and layer numbers across extensive areas.

## 19 Active Learning and Autonomous Microscopy

Deep kernel learning (DKL) extends structural analysis into active learning and automated 4D‐STEM experiments (Figure 10B) [120]. During acquisition, the model learns relationships between local structure and measured descriptors, then uses the predicted response and uncertainty to select subsequent measurement positions. In twisted bilayer graphene, this strategy reduced electron‐beam exposure while retaining information from boundaries and interfaces where strain is concentrated. Related demonstrations in MnPS_3_ have shown how adaptive sampling can reveal unexpected structural behavior while avoiding uniform irradiation of the full field of view.

These methods can also track dynamic processes at the atomic scale. This capability was demonstrated through analysis of defect evolution in WS_2_ during electron beam irradiation (Figure 10C) [136]. The framework combines convolutional neural networks with unsupervised clustering to rapidly classify thousands of lattice defects, resolving sulfur vacancy diffusion, and strain‐induced phenomena.

Machine learning has expanded analysis of strain effects in 2D materials, but several challenges persist. Integration of physics‐based constraints into ML frameworks remains essential, particularly for analyzing dynamic processes during STEM experiments. Future frameworks should combine the precision of supervised learning with the exploratory value of unsupervised methods while retaining physical constraints. Transfer learning and standardized data representations may help analysis frameworks accommodate changes in microscope settings, acquisition conditions, and sample preparation. Their usefulness will depend on transparent preprocessing/calibration and clear tests of whether a model trained on one material or instrument can be applied to another.

Key limitations include training‐set bias toward pristine samples, poor transferability across microscopes, and acquisition conditions, limited access to labeled 4D‐STEM and spectroscopy datasets, and uncertainty over whether learned features correspond to physically meaningful strain fields. These challenges are especially important for twisted and strained 2D materials, where small changes in acquisition conditions, sample preparation, beam exposure, or defect density can alter the measured structural response. Addressing these issues requires open datasets and transparent, physics‐aware model reporting that preserves symmetry and mechanical constraints. Additional hurdles include beam sensitivity and real‐time computational robustness. Resolving these limitations will be important for applying straintronics and twistronics to flexible electronics and quantum or energy‐efficient devices. Self‐driving microscopes are one possible outcome of this direction, but their usefulness will depend on reliable uncertainty estimates and physically meaningful acquisition rules.

Looking forward, machine learning may move from structural characterization toward inverse design of moiré superlattices. Emerging frameworks can search for strain configurations or twist angles associated with targeted electronic properties, including band gaps and Berry‐curvature distributions [138, 139]. Machine‐learned interatomic potentials also offer a route to simulating large‐period moiré supercells for which standard DFT is computationally prohibitive. Equivariant graph architectures such as NequIP and MACE can approach the accuracy of their training calculations within a defined configuration space while extending simulations to far larger atom counts [140, 141, 142, 143, 144]. Such models must report their training distribution, energy and force errors, out‐of‐distribution tests, and treatment of sliding, corrugation, defects, and high‐strain configurations before they can support predictive device design.

## 20 Summary and Future Perspectives

Straintronics and twistronics use lattice deformation and rotational alignment to tune structure‐property relationships in 2D materials. By modifying band structure and symmetry through interlayer coupling, these approaches enable phenomena such as flat‐band superconductivity, ferroelectric domains, tunable magnetism, and strain‐controlled optoelectronic response. Recent progress in growth, transfer, patterned substrates, piezoelectric actuation, and pressure‐based tuning has moved the field toward more reproducible control of strain and twist across increasingly complex heterostructures. Machine learning is further accelerating this transition through high‐precision strain mapping, automated structural classification, and inverse design of moiré superlattices.

Despite this progress, several challenges remain before strain‐ and twist‐engineered 2D materials can be translated into scalable technologies. Key limitations include twist‐angle disorder, nonuniform strain distributions, interfacial contamination, mechanical fatigue, environmental instability, and limited reproducibility across large‐area devices [6]. Addressing these challenges will require tighter feedback between synthesis, assembly, characterization, and modeling. As scalable fabrication and data‐driven design improve, straintronics and twistronics may evolve from exploratory research platforms into practical routes for reconfigurable devices and energy‐efficient quantum technologies.

## 21 Principles for Predictive Strain and Twist Engineering

For predictive design, nominal processing inputs must be separated from the local structure that the material actually adopts. A stated twist angle or applied strain is not sufficient by itself. Studies should report the spatial distribution of twist, heterostrain, stacking registry, corrugation, defects, and domain walls over the same region used for the relevant functional measurement. At small twist angles, the relaxed structure should be established experimentally or included in the model before the resulting states are interpreted.

Measurements should also be cross‐validated when one observable responds to more than one variable. Raman peak shifts, for example, can reflect strain, doping, temperature, defects, and substrate coupling, so correlation analysis or an independent structural probe is needed. Quantitative device metrics should remain tied to the material, layer number, geometry, temperature, field direction, strain state, and measurement protocol under which they were obtained. This prevents a device‐specific result from being presented as a universal material property.

The same discipline is needed for theory and machine learning. Multiscale models should state how the structure was relaxed, how parameters were obtained, and where the model is expected to fail, along with the measurements used for validation. ML studies should report the input, prediction target, training and test distributions, uncertainty, and out‐of‐distribution behavior. Large‐area growth and large‐area structural uniformity should be treated as separate achievements, because a continuous film does not necessarily possess a uniform twist angle, strain field, or reconstructed state.

## Conflicts of Interest

The authors declare no conflicts of interest.

## Data Availability

Data sharing is not applicable to this article as no new data were created or analyzed in this study.
